# Electronic Circular Dichroism of the Cas9 Protein and gRNA:Cas9 Ribonucleoprotein Complex

**DOI:** 10.3390/ijms22062937

**Published:** 2021-03-13

**Authors:** Monika Halat, Magdalena Klimek-Chodacka, Jagoda Orleanska, Malgorzata Baranska, Rafal Baranski

**Affiliations:** 1 Faculty of Chemistry, Jagiellonian University, Gronostajowa 2, 30-387 Krakow, Poland; monika.halat@uj.edu.pl (M.H.); jagoda.orleanska@doctoral.uj.edu.pl (J.O.); 2 Department of Plant Biology and Biotechnology, Faculty of Biotechnology and Horticulture, University of Agriculture in Krakow, AL. 29 Listopada 54, 31-425 Krakow, Poland; magdalena.klimek-chodacka@urk.edu.pl; 3 Jagiellonian Centre for Experimental Therapeutics (JCET), Bobrzyńskiego 14, 30-348 Krakow, Poland

**Keywords:** CRISPR/Cas9, DNA cleavage, ECD, spectroscopy, guide RNA, RNP complex, SpCas9

## Abstract

The *Streptococcus pyogenes* Cas9 protein (SpCas9), a component of CRISPR-based immune system in microbes, has become commonly utilized for genome editing. This nuclease forms a ribonucleoprotein (RNP) complex with guide RNA (gRNA) which induces Cas9 structural changes and triggers its cleavage activity. Here, electronic circular dichroism (ECD) spectroscopy was used to confirm the RNP formation and to determine its individual components. The ECD spectra had characteristic features differentiating Cas9 and gRNA, the former showed a negative/positive profile with maxima located at 221, 209 and 196 nm, while the latter revealed positive/negative/positive/negative pattern with bands observed at 266, 242, 222 and 209 nm, respectively. For the first time, the experimental ECD spectrum of the gRNA:Cas9 RNP complex is presented. It exhibits a bisignate positive/negative ECD couplet with maxima at 273 and 235 nm, and it differs significantly from individual spectrum of each RNP components. Additionally, the Cas9 protein and RNP complex retained biological activity after ECD measurements and they were able to bind and cleave DNA in vitro. Hence, we conclude that ECD spectroscopy can be considered as a quick and non-destructive method of monitoring conformational changes of the Cas9 protein as a result of Cas9 and gRNA interaction, and identification of the gRNA:Cas9 RNP complex.

## 1. Introduction

In 2020, the Royal Swedish Academy of Sciences has awarded the Nobel Prize in Chemistry for the development of a method for genome editing, as the consequence of discovery and research on Clustered Regularly Interspaced Short Palindromic Repeats (CRISPR) and CRISPR-associated (Cas) proteins. The CRISPR/Cas system is a fast developing technology allowing for genome editing, i.e., for generation of small genome modifications at defined sites of the target DNA or, in other words, for a precise, site-directed mutagenesis [1]. A wide range of potential applications and the simplicity, precision and effectiveness of genome editing also justified the earlier announcement of CRISPR/Cas the Breakthrough of the Year by Science journal in 2015 [2]. Despite editing approach, various Cas variants have been used to create chimeric molecules to elucidate protein interactions, and to activate or repress gene expression [3]. The CRISPR/Cas-based methods have been revolutionizing biological sciences, nowadays. Clinical trials of CRISPR-mediated gene therapy have been initiated [4]. Advanced research has been carried out for the application of CRISPR/Cas in human disease treatments, including AIDS, hemophilia, autism spectrum disorder, and cancer [5]. Additionally, several molecular diagnostic tools were invented for virus detection, pathogen identification, cancer mutation analysis or patient genotyping [6]. In plant sciences, genome-edited soybean of high nutritional value has been adopted to agriculture recently while other crops are awaiting commercialization [7,8].

**Figure 1:**
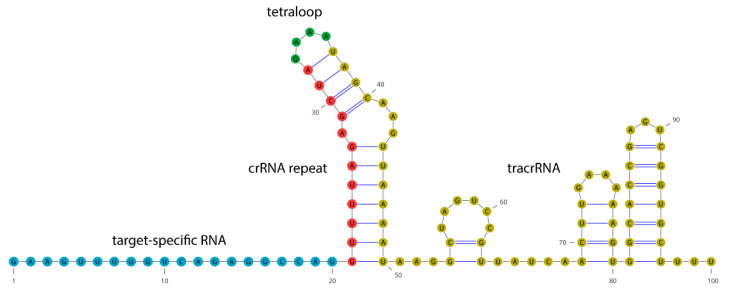
Single-guide RNA structure. In this work, the target-specific RNA sequence is complementary to the flavanone-3-hydroxylase (*F3H*) carrot (*Daucus carota* L.) gene (NCBI Acc. No. AF184270.1).

A native CRISPR/Cas system was discovered in microbes where it functions as an adaptive immune mechanism protecting against invading viruses [9]. The majority of prokaryotic genomes contain characteristic CRISPR locus with short (about 30 bp, on average) repeats interspaced by unique sequences of similar size [9,10,11]. These spacers are phage DNA fragments of variable sequences acquired by a microbe during past phage infections [12]. Each spacer and repeat pair are transcribed to CRISPR RNA (crRNA), which hybridizes with another RNA molecule, a small trans-activating crRNA (tracrRNA), to form a guide RNA (gRNA). Hence, gRNA is composed of the tracrRNA with a stem loop structure due to conserved nucleotide sequence and the crRNA molecule with 20 nt variable sequence at 5′ end that can hybridize to a complementary DNA strand. Cas protein, or usually a complex of various Cas proteins, forms a ribonucleoprotein (RNP) complex with gRNA, which guides the protein to the target phage DNA at the location with complementary sequence next to a short protospacer adjacent motif (PAM) of conserved, usually 3–6 nt sequence [13,14]. Then, Cas protein destroys phage DNA by its cleavage [15]. Further discoveries have paved the way for the utilization of this natural defense mechanism and have led to the development of CRISPR/Cas-based systems for DNA editing and research in eukaryotic organisms. In particular, it was found that about 100 nt long, single gRNA composed of crRNA and tracrRNA fragments linked with a tetraloop (Figure 1) can be used instead of a native hybrid gRNA [13]. In consequence, designing gRNA to target the desired DNA location as well as the delivery of gRNA to target cells was simplified and single gRNA has become routinely used.

**Figure 2:**
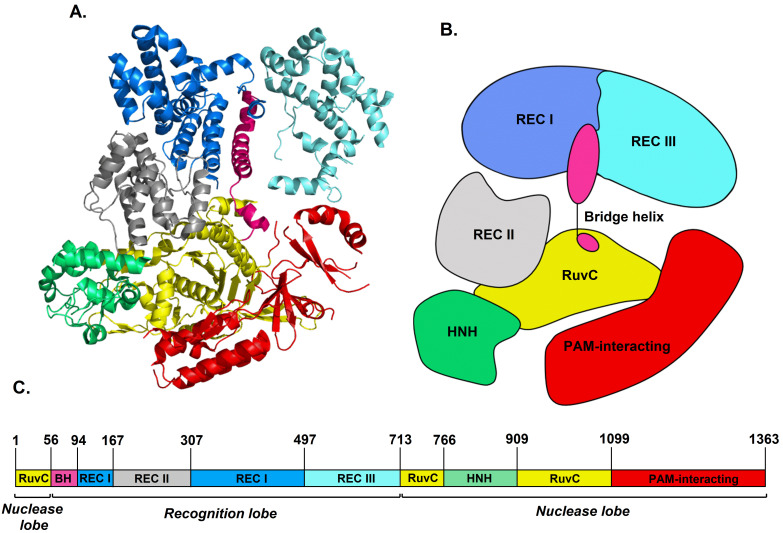
The structure of *Streptococcus pyogenes* Cas9 protein, which contains seven domains: Rec I, Rec II, REC III, Bridge Helix, RuvC, HNH, and PAM-interacting. Domains are presented in crystal (**A**), schematic (**B**), and map (**C**) form. Crystal image was rendered from RCSB PDB ID: 4CMP [19], using PyMOL software (Schrödinger Inc., New York, NY, USA).

Microbes have developed diverse CRISPR/Cas systems differing in the number and function of Cas proteins which often must form functional complexes capable of recognizing, binding, and cleaving phage DNA. However, single proteins performing all these functions have been identified [11]. The best characterized, and now commonly used for genome engineering, is the *Streptococcus pyogenes* Cas9 (SpCas9, pCas9) protein. Cas9 is a class II, type II, nuclease consisting of seven domains: REC I, REC II, REC III, HNH, RuvC, PAM-interacting, and bridge helix (Figure 2) [16]. The REC I domain (blue colour) is responsible for a connection with gRNA. In turn, the REC III domain (cyan colour) is used to sense the formation of RNP complex. The PAM-interacting domain (red colour) recognizes the PAM sequence at the target DNA. The arginine-rich bridge helix (pink colour) plays a key role in binding target DNA and modulates cleavage activity of the Cas9 protein [17]. Bot, HNH (green colour) and RuvC (yellow colour) domains are responsible for cleavage; each of them cleaves a single DNA strand 3 nt upstream of PAM that leads to a double-strand break [18,19]. The role of REC II domain (gray colour) has not been fully elucidated, yet, but it seems not critical for DNA cleavage [16,20].

The development of efficient CRISPR/Cas systems for genome editing or for other research purposes requires the selection of a proper Cas variant and designing correct gRNA. The ability of both components to form active RNP complex must be verified experimentally. Hence, a fast and non-destructive analytical method confirming RNP complex formation would be valuable. Here, we present an electronic circular dichroism (ECD) study on the Cas9 protein in its native form and bounded with gRNA. ECD spectroscopy is based on a differential absorption of left- and righthanded circularly polarized light in the UV-Vis range by chiral molecules. It is widely used to monitor structural changes of organic compounds or important macromolecules in solution [21,22] as nucleic acids [23,24] and proteins, especially to estimate the secondary structure of proteins including α-helix, β-sheet, or random coil [25,26,27]. Proteins give strong ECD signals in the far-UV region, dominated by the nπ* (~220 nm) and ππ* (~190 nm) transitions of amide groups, which are influenced by the geometries of the polypeptide backbones and reflective of the different types of secondary structures. In contrast to the other techniques like X-ray crystallography and NMR, ECD does not require much time and specific condition of preparing a sample [28]. Thus, a good quality ECD spectrum can be obtained for a quite low concentration of protein, in neutral water environment and with a short acquisition time (in 30 min or less). Hence, ECD measurement, possibly supported by theoretical calculations, is a powerful tool to examine the structure of proteins [28,29]. What is more, there are only a few reports engaging ECD spectroscopy, in a very little extent, to analyse CRISPR/Cas systems [30,31,32,33]. Two of them concern the Cas9 protein specifically [30,32]. So, we found that ECD can be considered as a quick and effective method to identify the gRNA:Cas9 RNP complex. To confirm this approach is non-destructive to the protein and its complex with RNA, we induced also in vitro DNA cleavage to show the biological activity of RNP after ECD measurements.

## 2. Results

### 2.1. Circular Dichroism of Cas9 Protein and gRNA

**Figure 3:**
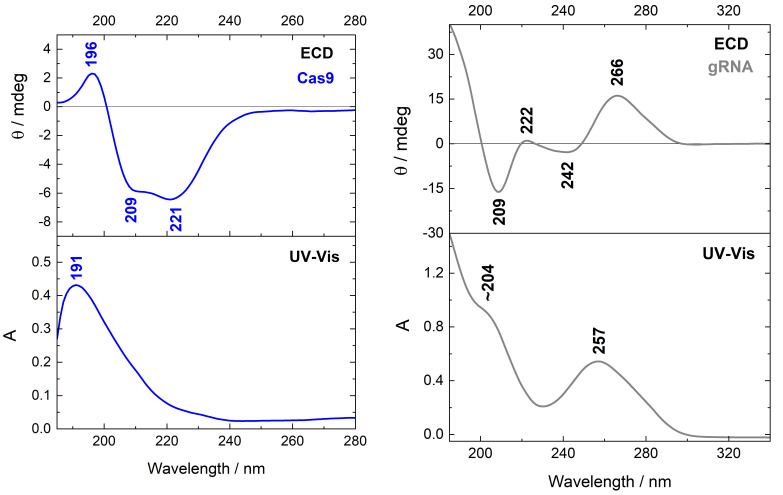
ECD and UV-Vis spectra of Cas9 protein (blue line, C = 9.35 × 10^−7^ M) and gRNA (gray line, C = 1.07 × 10^−5^ M).

To determine secondary structure of Cas9 protein, ECD measurements were performed. Recorded ECD and UV-Vis spectra are presented in Figure 3 (blue line). Proteins give strong ECD signals in the range of 185–300 nm, which carry information about their secondary structure. For Cas9, ECD profile reveals two negative and one positive pattern located at 221, 209, and 196 nm, respectively. Intensities of negative bands are slightly different, as indicated by the calculated ratio [θ]_221_/[θ]_209_ = 1.12. In addition, these bands are much more intense than the positive ones. In turn, the maximum of UV-Vis absorption for Cas9 protein is located at 191 nm (Figure 3).

The ECD spectrum registered for gRNA is also shown in Figure 3 (gray line). Going from longer to shorter wavelengths, the spectral profile exhibits positive/negative/positive/negative pattern of ECD bands situated at 266, 242, 222 and 209 nm, respectively. The UV-Vis spectrum of gRNA reveals maximum at 257 nm and a shoulder at around 204 nm.

### 2.2. Circular Dichroism of the RNP Complex

**Figure 4:**
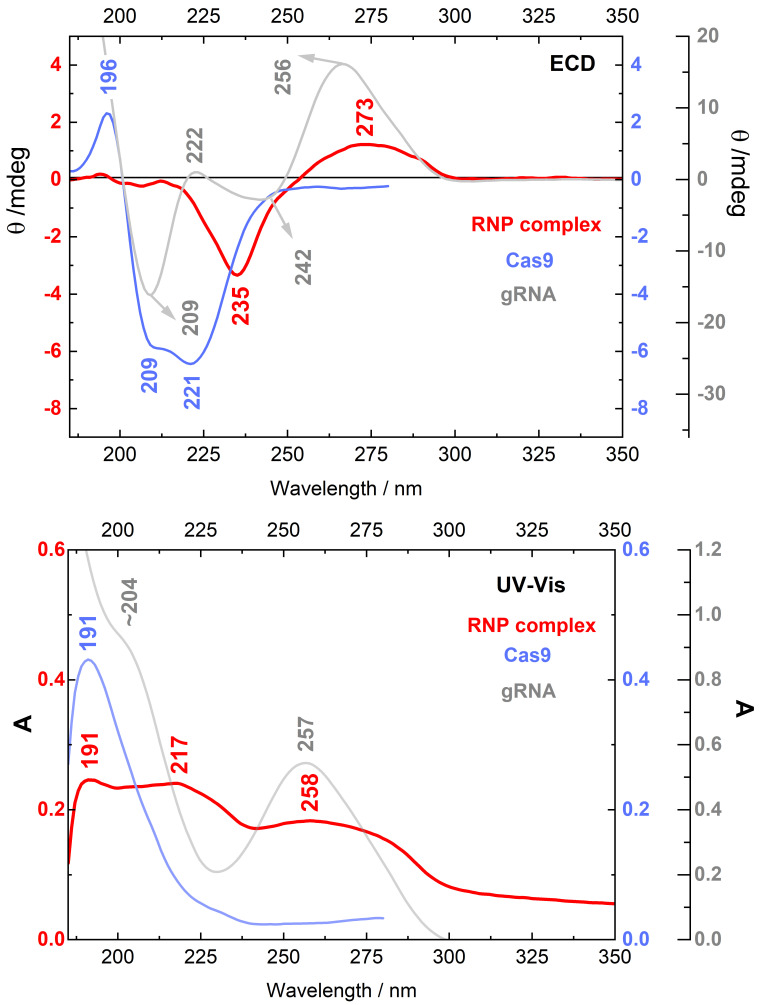
ECD and UV-Vis spectra of the Cas9:gRNA complex (red line and left scale; in the 1:1 molar ratio with individual concentrations C_CAS9_ = 9.98 × 10^−6^ M and C_gRNA_ = 1.07 × 10^−5^ M) in comparison to the spectra of the Cas9 protein (light blue line and the 1st right scale, C = 9.35 × 10^−7^ M) and gRNA (light gray line and the 2nd right scale C = 1.07 × 10^−5^ M). ECD: electronic circular dichroism.

To find an effective and quick method for identification of the gRNA:Cas9 complex, ECD measurements were performed also for the RNP complex solution. Collected ECD and UV-Vis spectra are presented in Figure 4. The RNP ECD profile (red line) shows the positive/negative pattern of the bands located at 273 and 235 nm. The intensities of these bands are approximately one order in magnitude lower than the one in the spectrum of Cas9 protein (blue line). Moreover, the ECD spectrum of RNP complex differs significantly from the ECD spectrum of unassociated gRNA (light gray line). Both spectra have similar positive/negative pattern of two following bands going from longer to shorter wavelengths. However, those ECD bands have different positions and are shifted in relation to each other. The ECD spectrum of gRNA reveals also additional bands situated at 222 and 209 nm.

The UV-Vis spectrum of the gRNA:Cas9 complex shows tree maxima, observed at 258, 217 and 191 nm, two of which can be assigned to gRNA (258 nm) and protein (191 nm), and the band at 217 nm cannot be assigned to any of the components.

### 2.3. Biological Activity of the Cas9 Protein and RNP Complex

**Figure 5:**
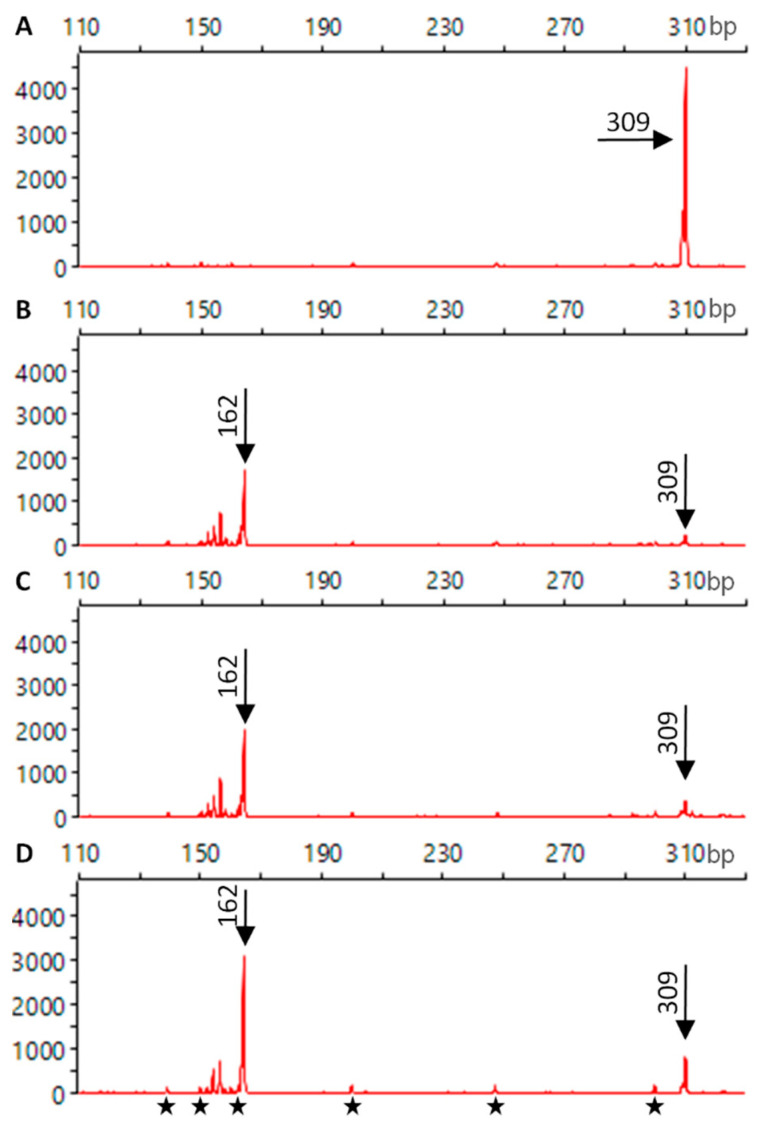
Electropherograms of ROX-labelled DNA fragments detected by a capillary electrophoresis. Untreated DNA of 309 bp in length (**A**), DNA incubated with the untreated RNP complex—control (**B**), DNA incubated with the RNP complex containing ECD-measured Cas9 (**C**), DNA incubated with the ECD-measured RNP complex (**D**). Stars—GeneScan™ 500 LIZ size marker.

The Cas9 biological activity after ECD measurements was verified by inducing DNA cleavage in vitro and the sizes of the obtained ROX-labelled DNA fragments were determined using capillary electrophoresis. Fluorescent signals from untreated sample revealed the presence of DNA, which size was exclusively 309 bp (Figure 5A). The same sample was incubated with the RNP complex, which was not used for ECD measurements (control). After incubation, fluorescent signals were obtained predominantly from DNA fragments of 162 bp in length (Figure 5B). Weaker signals from shorter (150–161 bp) fragments were also present while the 309 bp fragment signal was very weak. The presence of 162 bp or shorter fragments indicated that almost all DNA was cleaved by the control RNP complex.

The ECD measurements of Cas9 protein were performed and then such protein was used for RNP complex formation. Independently, a new RNP complex was prepared by incubating unused gRNA and Cas9, and then such RNP complex was subjected to ECD measurements. Both complex types, the RNP complex containing ECD-measured Cas9 and ECD-measured RNP complex, were used for DNA cleavage in vitro to check their biological activity. For both samples, electropherograms showed fluorescent signals indicating the presence of short DNA fragments of the same size as in the control, i.e., predominantly 162 bp fragments (Figure 5C,D).

## 3. Discussion

### 3.1. Cas9 and gRNA:Cas9 Complex Structures Revealed by ECD Spectroscopy

Based on ECD spectroscopy, a secondary structure of protein like α-helix, β-sheet or random coil can be identified [27,28]. Excitation of a number of closely connected chromophores (peptide bonds), being in a regular spatial disposition in protein, gives rise to characteristic spectral features below 240 nm. The experimental ECD spectrum of Cas9 protein (Figure 3, blue line) shows negative/positive pattern, like α-rich proteins, with two negative bands of nearly the same intensity, observed at 221 and 209 nm, accompanied by the positive one at 196 nm. The origin of these ECD bands is assigned to the nπ*, ππ*ǁ (parallel to the helix axis) and ππ*⊥ (perpendicular to the helix axis) electronic transitions of the peptide bond, respectively [27,34], where n is the nonbonding molecular orbital, π is the HOMO level, while π* refers to the LUMO state [34]. In contrast to a typical α-helix ECD spectrum [35], the positive Cas9 band is slightly red-shifted (192→196 nm) and shows twice-lower intensity than negative ones, which in consequence shows an impact of the remaining secondary structures in the protein. According to the crystallographic data, the SpCas9 molecule adopts a crescent shape with approximate dimensions of 100 Å × 100 Å × 50 Å [19]. Its secondary structure is determined by α-helices with about a twice-less content of stranded antiparallel β-sheets that, in turn, are the most prevailing in the PAM-interacting and RuvC domains [36] (Figure 2A). Moreover, using an interactive interface to the STRIDE program [37] presented in detail by Frishman and Argos [38], the percentage content of individual secondary structure can be estimated based on knowledge of the atomic coordinates of proteins. Thus, according to calculations based on the STRIDE program, beyond the main contribution of α-helix (49.6%), the SpCas9 protein also contains: 4% of 3_10_-helix, 9.4% of β-sheet, 1.6% of β-bridge and 17% of β-turn classified as I, I′, II, II′, VIa and VIb [39]. The remaining of 18.6% is a random coil structure. Hence, the decrease in intensity, as well as the position of positive band at 196 nm in the Cas9 ECD spectrum may be attributed to the presence of 3_10_-helix, what was theoretically predicted by Manning and Woody [40]. The 3_10_-helix is a relatively common structural element in globular proteins (3%), occurring often at the ends of α-helices [41,42]. The average conformational parameters of both helices are rather close to each other, but the 3_10_-helix is gently tighter and more elongated, as well as is characterized by the different C=O∙∙∙H−N intermolecular hydrogen bonding scheme [41,43]. Then, Toniolo et al. [44] reported the experimental ECD spectrum of 3_10_-helix, confirming the positive band of very weak intensity near 195 nm. However, as opposed to the theoretical calculations, another negative strong maximum at 184 nm, not seen here (Figure 3, blue line), was also registered. The 3_10_-helix seems to have a positive signal around 190 nm of an uncertain magnitude [45]. Therefore, it cannot be definitively determined whether the presence of the 3_10_-helix in Cas9 protein contributed to the intensity reduction of the positive Cotton effect, and a higher contribution of β-sheet than one might expect cannot be ruled out.

Certainly, the β-sheet content has also an impact on the Cas9 ECD profile, what is observed in the spectra of α/β-rich proteins like ribonuclease A (21% α, 33% β) or subtilisin (30% α, 18% β) [45]. Signals of α-helix are stronger than β-sheet ones, so proteins with comparable amount of both secondary structures generally show the qualitive spectral features of the α-helix keeping the same negative/positive ECD profile. Although some differences in the intensity or localization of ECD bands are present, especially as a decrease in intensity of positive band around 195 nm in ribonuclease A spectrum [45]. Significant effect on protein ECD pattern may have also β-turns [45,46], enabling reversal of the polypeptide chain direction. These motifs constitute as the third most common secondary structural elements occurring in proteins. For example, the type I reveals α-helix-like ECD spectrum, while the type II shows a β-sheet-like one, what was confirmed experimentally [47]. The Cas 9 has β-turn motifs in its structure, what may be manifested in reduced intensity of the positive band at 196 nm, as well as in a gentle difference in intensity between negative ECD bands ([θ]221/[θ]209 = 1.12).

In contrast to ECD spectroscopy, UV-Vis spectra of different proteins secondary conformations do not reveal specific features, besides slight shift of the main electronic transition. The Cas9 UV-Vis spectrum (Figure 3, blue line) has only one maximum at 191 nm that is typical for pure α-helix structure and comes from the ππ* excitation in the amide group [48].

Like proteins, the absorption spectra of nucleic acids are dominated by ππ* electronic transitions, which are all polarized in the plane of the bases. Moreover, both wavelength maxima and transition intensities vary depending on the base sequence and structure adopted [48]. Nonbonding electrons of the amide-like oxygens can be also excited to π* orbitals; however, nπ* transitions are characterized by low intensity and are usually buried by the intense ππ* ones [49]. Here, we observe maximum of UV-Vis gRNA band at 257 nm (Figure 3, gray line), which can be assigned to ππ* excitation and corresponds to the literature data recorded for various polynucleotides [48]. In turn, the experimental ECD spectrum of gRNA (Figure 3, gray line) shows positive/negative/positive/negative pattern with bands observed at 266, 242, 222 and 209 nm, respectively, what is typical for the A-type helix geometry assigned to RNA duplexes [50,51]. Thereby, it confirms that gRNA has the repeat and anti-repeat regions adopting the A-form-like conformation [52]. In general, RNA is formed by canonical double-helical fragments, along with non-canonical secondary structure motifs like internal loops, hairpins, bulges, and the most widespread ones: single mismatches [53]. All these motifs play relevant functions in providing binding sites for proteins, small molecules, or nucleic acids, and have impact in folding the correct tertiary and quaternary structures. Theoretical calculation shows that ECD signal of polynucleotides mainly origins from the exciton coupling interaction between bases stacked in asymmetric structure of higher ordering like helices, than from the base-sugar interaction in monomeric nucleotides, as well as it arises for the most part from the ππ* transitions, rather than nπ* ones [54]. Thus, perturbation of a nucleic acid ECD profile, that appears due to binding of a protein, generally reflects changes in short-range base–base interactions, providing confirmation for transitions between secondary structures (A↔B, B↔Z) or alternations in base pairing, base stacking, or bending of a DNA or RNA duplex [55].

Despite a lot of ECD studies describing protein–-nucleic acids interactions [55,56], here we present for the first time the experimental ECD spectrum of gRNA:Cas9 RNP complex (Figure 4, red line). This unique RNP hybrid reveals positive/negative ECD pattern with bands located at 273 and 235 nm, which differs significantly from individual spectral profiles of each macromolecule. The Cas9 upon gRNA binding changes dramatically its structure through wide intermolecular rearrangements. According to the literature, one of the most prominent changes takes place in the REC III domain, which moves ∼65 A˚ toward the HNH domain due to gRNA connection [20], what is definitely reflected by the characteristic ECD spectral profile of RNP hybrid in comparison to the ECD spectrum of a free protein (Figure 4, light blue line). Moreover, using the STRIDE server [37] and the crystallographic data PDB ID 4ZT0 [57], the percentage content of individual secondary structure in active RNP complex can be provided: 41.5% of α-helix, 4.5% of 3_10_-helix, 10.7% of β-sheet, 2% of β-bridge, 22.4% of β-turn and 18.8% of random coil structure. Based on presented data, it can be concluded that the Cas9 changes its secondary structure elements upon gRNA binding, mainly through loss of α-helicity (49.6→41.5%), and slight increase in the content of β-turns (17→22.4%). However, it is hard to determine those changes from the RNP ECD profile, which has no representative spectral features for still-prevalent α-helix structure. A major change in α-helical content undergoes also among a DNA binding proteins like transcription factors, which have been mostly studied among proteins by ECD below 250 nm [55]. Unlike the gRNA:Cas9 complex, transcription factors exhibit an enhancement of α-helix structure in the bound state around 10–15%. However, in some cases like recombination activation gene 1 protein (the RAG1) [58] and human translin protein [59], a strong decrease in the protein α-helicity after nucleic acids complexation was confirmed. On the other hand, the ECD spectrum of RNP complex is strongly red-shifted and do not reveal any characteristic bands below 220 nm, which can be assigned to any of the protein secondary structures. In addition, its ECD signals do not coincide with the gRNA spectrum indicating that the polynucleotide chain adopted also different geometry due to connection with the Cas9 protein. What is interesting, there are some similarities in ECD patterns between the gRNA:Cas9 complex and chromatin [60], especially in the range 240–300 nm that is practically the same in both cases. The ECD spectrum of the complex between EsColi ribonuclease and RNA also bears some resemblance to the RNP [61]. However, the RNP ECD profile is very individual and it is hard to compare with literature results obtained for other protein–nucleic acid hybrids. The UV-Vis spectrum of gRNA:Cas9 (Figure 4, red line) differs somehow from those of Cas9 and gRNA, but it includes absorption bands characteristic of each macromolecular component and does not allow to monitor changes in secondary structure geometry.

### 3.2. Cas9 Binds gRNA and Retains Cleavage Activity after ECD Measurements

We have previously demonstrated that carrot genome can be edited by delivering *S. pyogenes* Cas9 and gRNA expression vectors to living cells which led to the RNP complex formation in vivo [62]. The gRNA was designed to target the 20 nt sequence of the flavanone-3-hydroxylase (*F3H*) gene located upstream the GGG PAM sequence. Sequencing analysis revealed that the target DNA was cleaved usually 3 bp upstream of PAM, although small nucleotide deletions in close proximity were also identified in some samples.

In this work, we have verified that the measurements using ECD spectroscopy do not affect Cas9 biological activity by performing in vitro DNA cleavage experiments. Verification of RNP complex activity using in vitro cleavage has been reported earlier and such approach has several advantageous over experiments with living cells: it is fast, performed in controlled conditions, and the results are not affected by complex cell processes [63]. For that purpose, we assembled the above said gRNA molecules and obtained their complexes with SpCas9 to carry out in vitro cleavage of DNA utilizing a laboratory-amplified fragment of the *F3H* carrot gene as the target. The used gRNA was designed to guide Cas9 protein to the 309 bp DNA and to enable DNA cleavage at the selected target site to two, 162 bp and 147 bp, fragments. A fluorescent capillary electrophoresis was employed to confirm cleavage due to its high accuracy [64]. As the used DNA was ROX-labelled at only one end, the fluorescent signals registered during a capillary electrophoresis could originate from the unmodified DNA molecules and 162 bp cleaved products. The in vitro DNA cleavage and detection of predominantly 162 bp products confirmed the activity of control RNP complex, as expected.

Using the same approach, two other RNP complexes were also checked for their biological activity. In contrast to the control, either the whole RNP complex was subjected to ECD measurement before in vitro DNA cleavage experiments or only the Cas9 protein was measured prior to the RNP complex formation, and then this complex was measured using ECD. In all samples, the expected 162 bp cleaved fragments were identified. No essential difference in the activity of RNP complexes was found nor their activity differed from the control. Hence, in vitro DNA cleavage confirmed that: (1) gRNA was properly designed and synthesized, (2) Cas9 protein was able to form complex with gRNA, (3) gRNA properly directed Cas9 to the target DNA site, and (4) Cas9 had cleavage activity. Moreover, the same conclusions apply to the ECD-measured Cas9 protein used for RNP complex formation. The ECD measurements did not restrict Cas9 protein ability to interact with gRNA and DNA nor its enzymatic activity. Furthermore, ECD measurements of the already formed RNP complex also did not affect these properties. Hence, it can be concluded that after ECD measurements Cas9 retains its structure capable to form an active complex with gRNA to efficiently cleave DNA.

The obtained results provide new opportunities for analytical validation of correct gRNA designing. The ECD spectroscopy can be used to confirm the presence of RNP complexes in the sample without adverse effects on their biological activity. This is in particular valuable in case of unsuccessful DNA cleavage performed in vivo or in vitro. As indicated above, several requisites must be fulfilled for efficient DNA cleavage by RNP complex [13,16,20]. Therefore, the lack of cleaved products may be due, inter alia, to aberrant interaction of gRNA with the Cas protein. The detection of such events would be valuable in research aiming in verification of gRNA differing in their structure. In particular, the Cas activity depends on the gRNA length and tracrRNA fragment structure [65,66]. Also new Cas protein variants have been reported and they differ in structure which, in turn, determines the structure of interacting gRNA [3,13]. Hence, complementing experimental and bioinformatic approaches to studying RNP complexes [67], ECD spectroscopy can help reveal Cas–gRNA compatibility in a fast and simple fashion.

## 4. Materials and Methods

### 4.1. Cas9 Protein

*Streptococcus pyogenes* Cas9 (EnGen^TM^ Spy Cas9 NLS) protein was purchased from New England Biolabs Inc. (Ipswich, MA, USA) as a buffer solution (300 mM NaCl; 10 mM Tris-HCl; 0.1 mM EDTA; 1 mM DTT; 50% glycerol) with a protein concentration of 3.22 mg/mL (100 μL; 2000 pmol).

### 4.2. gRNA Design and Transcription

The 20 nt target-specific RNA sequence of single-guide RNA (Figure 1) was designed to target the second exon in the flavanone-3-hydroxylase (*F3H*) carrot (*Daucus carota* L.) gene (NCBI Acc. No. AF184270.1) at 632–651 position (GAAGTTTTGTCAGAGGCCAT) using the CasOT software [68]. The complementary DNA oligonuclotide was synthesized commercially (Genomed, Warsaw, Poland). This fragment was assembled with oligonucleotides of the tracrRNA part of the gRNA using the GeneArt™ Precision gRNA Synthesis Kit (Invitrogen, Carlsbad, CA, USA), and the final product was transcribed to the gRNA molecules according to the kit manufacturer’s recommendations. The correct assembly and nucleotide sequence of gRNA was confirmed by sequencing (Genomed, Warsaw, Poland).

### 4.3. Formation of the gRNA:Cas9 RNP Complex

The synthesized gRNA was mixed with Cas9 in 1:1 molar ratio and incubated at 37 °C for 20 min in order to form the RNP complex. In detail, 4.24 μL of the 7.58 mg/mL gRNA solution was added to 50 μL of the 3.22 mg/mL Cas9 solution. The obtained mixture was vortexed and then incubated. The RNP complex was used either for ECD measurement or for in vitro DNA cleavage.

### 4.4. UV-Vis/ECD Measurements

Samples of Cas9 protein, gRNA and RNP complex were measured by means of UV–Vis/ECD spectroscopy using JASCO J-815 spectrometer (JASCO Corporation, Tokyo, Japan). To avoid supersaturation of the detector, all samples were diluted with very clear nuclease-free water. The concentration of the individual solutions was: 9.35 × 10^−7^ M for the Cas9 protein, 1.07 × 10^−5^ M for the gRNA in both single and RNP form, as well as 9.98 × 10^−6^ M for the Cas9 nuclease in RNP complex. All UV–Vis/ECD spectra were recorded with the use of 0.1 cm path length quartz cuvette, as well as with the following settings: 185–350 nm spectral range, 100 nm/min scanning speed, 1 nm bandwidth, 0.2 nm step size, 0.5 s response time and 10 scans of accumulation. Finally, baseline and solvent corrections were done with the JASCO software (JASCO Corporation, Tokyo, Japan).

### 4.5. DNA Amplification

Two HPLC purified primers (F: 5′-GCAAGATTGGCGAGAGATAG-3′ and R: 5′-AGCAAGAGCGTAATTGTGCC-3′) designed for the amplification of 309 bp DNA fragment of the second exon in the *F3H* carrot gene (NCBI Acc. No. AF184270.1) during polymerase chain reaction (PCR) were ordered from Genomed company (Warsaw, Poland). The F primer was labelled with the ROX fluorescent dye at its 5′ end. The PCR reaction mixture set up in 20 µL volume contained 0.1 µM of each primer and a commercial 2× buffer composed of Taq polymerase and dNTPs (PCR Mix Plus, A&A Biotechnology, Gdynia, Poland). The reaction was carried out in the Eppendorf Mastercycler thermocycler (Eppendorf, Hamburg, Germany) with the following conditions: the initial denaturation at 94 °C for 4 min, 35 cycles of 45 s at 94 °C, 30 s at 60 °C, and 60 s at 72 °C, and the final extension at 72 °C for 5 min.

### 4.6. In Vitro DNA Cleavage

The RNP complex, in the amount of 9 µmol Cas9 and 9 µmol gRNA, was mixed with 0.9 µmol of the PCR amplified 309 bp DNA fragments, which were earlier purified on a column (Wizard^®^ SV Gel and PCR Clean-Up, Promega, Madison, WI, USA). The final reaction mixture contained 10xNEBuffer 3.1 (100 mM NaCl, 50 mM Tris-HCl, 10 mM MgCl_2_, 100 µg/mL BSA) adjusted to 30 µL volume in RNase free water. DNA cleavage was performed at 37 °C for 15 min followed by 10 min incubation at RT, after the addition of 1 µL of Proteinase K (Sigma-Aldrich, St. Louis, MO, USA). The DNA fragments were separated using a capillary electrophoresis (3730XL DNA Analyzer, Applied Biosystems, Foster City, CA, USA) combined with the detection of fluorescent dyes. DNA fragment sizes were determined using the GeneScan™ 500 LIZ size marker (Thermo Fisher Scientific, Waltham, MA, USA) and visualized using the PeakScanner v.2.0 software (Thermo Fisher Sci., Waltham, MA, USA).

## 5. Conclusions

In this work, characteristic spectra of the Cas9 protein, gRNA, and their complex were registered using ECD spectroscopy. In particular, the RNP complex showed a distinctive spectral pattern resulting from some structural rearrangements in the Cas9 protein. The formation of RNP complex as well as its activity is determined by the structure of Cas protein and interacting gRNA molecule. Hence, we show that ECD spectroscopy can be used for verification of the Cas9 ability to bind specific gRNA and for identification of the successfully formed RNP complex. Moreover, the activity of the RNP complex was further confirmed by conducting the in vitro DNA cleavage. The results show that either Cas9 protein or RNP complex retain their biological activities after the ECD measurements, and they are able to bind and cleave the target DNA. The experiments have shown the potential of the ECD in structural studies of various Cas proteins and their interactions with specific gRNAs, in an undoubtedly non-destructive manner. To facilitate the research and use of continuously and fast evolving CRISPR/Cas-based systems in biological sciences, reliable and simple techniques allowing characterization of the CRISPR/Cas individual components are needed, and ECD can be considered as such.

## Data Availability

Publicly available datasets were analyzed in this study. This data can be found in the RCSB Protein Data Bank here: https://www.rcsb.org/structure/4CMP and https://www.rcsb.org/structure/4ZT0.
